# Prevalence of Anti-*Anisakis simplex* Antibodies in a Cohort of Patients with Inflammatory Bowel Disease in Norway

**DOI:** 10.3390/pathogens14080769

**Published:** 2025-08-04

**Authors:** María P. de la Hoz-Martín, Juan González-Fernández, Juan Carlos Andreu-Ballester, Marte L. Hoivik, Petr Ricanek, Torunn Bruland, Arne K. Sandvik, Carmen Cuéllar, Ignacio Catalán-Serra

**Affiliations:** 1 Unidad de Parasitología, Departamento de Microbiología y Parasitología, Facultad de Farmacia, Universidad Complutense de Madrid, 28040 Madrid, Spain; juangonzalez@ucm.es; 2 Fundación FISABIO—Salud Pública de Valencia, 46020 Valencia, Spain; jcandreuballester@outlook.com; 3 Department of Gastroenterology, Oslo University Hospital, 0424 Oslo, Norway; marte.lie.hoivik@gmail.com; 4 Department of Gastroenterology, Division of Medicine, Akershus University Hospital, Lørenskog, and University of Oslo, 1478 Oslo, Norway; petr.ricanek@medisin.uio.no; 5 Department of Clinical and Molecular Medicine, Norwegian University of Science and Technology, 7491 Trondheim, Norway; torunn.bruland@ntnu.no (T.B.); arne.sandvik@ntnu.no (A.K.S.); ignacio.catalan@ntnu.no (I.C.-S.); 6 Department of Gastroenterology, Levanger Hospital, Nord-Trøndelag Hospital Trust, 7600 Levanger, Norway

**Keywords:** *Anisakis simplex*, inflammatory bowel disease, ulcerative colitis, Crohn’s disease

## Abstract

This study assessed the seroprevalence of anti-*Anisakis simplex* antibodies in Norwegian patients with inflammatory bowel disease (IBD), specifically ulcerative colitis (UC) and Crohn’s disease (CD), compared with healthy controls. Associations between anti-*A. simplex* antibody positivity and clinical or laboratory parameters in IBD were also explored. A total of 86 UC patients, 68 CD patients, and 41 healthy controls were prospectively enrolled from four Norwegian hospitals (2013–2022). Diagnosis and disease activity were established using standard clinical, endoscopic, and biomarker criteria. Serum samples were analyzed for total Ig, IgG, IgM, IgA, and IgE antibodies against *A. simplex* and *Pseudoterranova decipiens* using ELISA. Anti-*A. simplex* IgG seroprevalence was 4.9% in controls and 3.2% in IBD (3.5% UC, 2.9% CD). IgM seroprevalence was 0% in all groups. IgA seroprevalence was higher in IBD (16.2%) than controls (4.9%), with 14.0% in UC and 19.1% in CD. IgE seroprevalence was low across all groups. Smoking correlated with lower antibody levels and higher surgery rates. In UC, higher anti-*A. simplex* IgG and IgE levels were associated with milder disease and better prognosis. Anti-TNFα and azathioprine treatments were linked to higher anti-*A. simplex* IgA. Norwegian UC and CD patients had significantly higher anti-*A. simplex* total Ig and IgA seroprevalence than healthy controls, indicating increased exposure or immune response. Anti-*A. simplex* IgG and IgE may serve as markers of clinical activity in UC. Further research is warranted to clarify the clinical significance of these findings.

## 1. Introduction

Inflammatory bowel disease (IBD) is a chronic inflammatory disease of the gastrointestinal tract with systemic involvement. The term IBD is most often used to describe two distinct conditions: ulcerative colitis (UC) and Crohn’s disease (CD) [1].

In recent decades, there has been an increase in the incidence of these diseases, the cause of which remains unknown. Both the incidence and prevalence of IBD in Norway are among the highest in the world. In 2017, the incidence of IBD was 40 (CD: 14.6, UC: 25.7) per 100,000 person-years, and the prevalence was 0.77% (CD: 0.27%, UC: 0.50%) [2]. The peak age for IBD occurrence is 20–30 for CD and 30–40 for UC [3]. These patients experience periods of remission and flares and suffer from complications such as hospitalization, and surgery, which have a high impact on quality of life [4].

Ulcerative colitis (UC) is characterized by lesions in the colonic mucosa. Symptoms include persistent diarrhea accompanied by visible blood and mucus, tenesmus, and lower abdominal pain. Crohn’s disease (CD) can affect any part of the digestive tract with lesions involving all layers of the intestinal wall, which can lead to the formation of fistulae, abscesses, and perforations. Abdominal pain, chronic diarrhea with or without blood in stool, weight loss, fatigue and fever are the most common symptoms [5].

Currently, the etiopathogenesis of these diseases is not fully understood. The origin of these diseases is considered to be multifactorial, involving genetic factors, environmental factors, the composition of the flora, and the immune response [6]. The hygiene hypothesis proposes that increasing hygiene measures and decreasing exposure to certain organisms could contribute to the development of autoimmune diseases due to the alteration of the immune system. This is a tentative explanation for the increasing prevalence of IBD in newly industrialized countries [7]. Furthermore, multiple studies have demonstrated the importance of the intestinal microbiota and its relationship with the immune status, highlighting the immunomodulatory effect of IgA [8,9,10]. Likewise, patients with IBD have altered SIgA1 and SIgA2 levels [11].

The use of immunosuppressive drugs, altered immune status, and chronic damage to the intestinal barrier increase susceptibility to opportunistic infections [12], and we previously observed that CD patients are a group at risk for microsporidiosis [13].

*Anisakis simplex* is a nematode parasite that can invade the stomach or intestinal wall in humans [14]. There are also described cases in which intestinal infection by *Anisakis* has been confused with CD [15,16,17]. In addition, intestinal anisakiosis and CD are similar in some aspects, such as the presence of granuloma in the biopsies and their preferred ileal location. On the other hand, mucosal damage in IBD increases the number and size of paracellular pathways and permeability [18], which in turn contributes to increased exposure to antigens of gut-associated lymphoid tissues [19].

Previous studies have shown a high prevalence of specific anti-*A. simplex* antibodies in IBD, observing a relationship between the presence of antibodies against the parasite antigens and T cell subsets, especially γδ in CD patients [16,20,21]. However, the clinical significance of this finding is uncertain. A possible explanatory hypothesis is that in patients with IBD, an alteration in innate immunity, such as a decrease in γδ T cells in the peripheral blood and intestinal mucosa of patients, could create a state of immunosuppression that would facilitate sensitization by *A. simplex*. Boland et al. [22] demonstrated, via single-cell and deep learning image analysis, significant enrichment of specific γδ T cell subsets in UC blood. Intraepithelial γδ T cells were reduced in inactive UC versus CD, despite elevated CD3+ T cells. Disease activity correlated with γδ T cell reduction in UC, highlighting phenotype-specific immunological roles [23]. Likewise, Chen et al. [24] identified several potential lymphocyte subset-related predictors of IBD progression and treatment response.

Helminth infections exhibit dual immunomodulatory roles, suppressing autoimmune diseases via Th2 and regulatory T-cell responses that downregulate proinflammatory cytokines such as IFN-γ and IL-17 [25,26]. However, *Anisakis* extracts can induce inflammation in human colonic cells by elevating COX-2 and IL-6/IL-8, highlighting context-dependent effects [27]. Therapeutic potential thus hinges on specific helminth species and host interactions [25,28].

Following our line of research in relation to the deficiency of γδ T cells in CD patients [21,29], we propose the working hypothesis that a parasite such as *A. simplex*, frequently ingested through fish consumption, could take advantage of the state of altered immunity in IBD to infect these patients more frequently than the healthy population. Our main objective is to characterize a large Norwegian population of IBD by the presence of antibodies against *A. simplex* and compare the results with data from healthy controls.

The possible relationship between positivity for anti-*A. simplex* antibodies and different clinical and laboratory parameters in IBD was also studied. In addition, we aim to provide new data on the seroprevalence of anti-*A. simplex* antibodies in the healthy Norwegian population to explore serological associations and immune profiles in the context of parasitic exposure and distinct inflammatory activity.

## 2. Materials and Methods

### 2.1. Study Population

All study subjects were included prospectively from individuals referred for colonoscopy to the outpatient clinic at the Department of Gastroenterology and Hepatology, St. Olav’s University Hospital in Trondheim, Levanger Hospital, Kristiansund Hospital and at Molde Hospital in the Central Norway Regional Health Authority (Norway) between 2013 and 2022. Biologic samples were consecutively stored in a cross-sectional IBD biobank at the Norwegian University of Science and Technology (NTNU), Trondheim.

The diagnosis of IBD was based on standard clinical, endoscopic, radiological, and histological criteria. Clinical information and disease extent were recorded at the time of inclusion. Clinical disease severity was determined based on the Mayo clinical scoring for UC and the Harvey–Bradshaw index (HBI) for CD. Endoscopic mucosal disease activity was reported as the Mayo endoscopic score for UC and Simple Endoscopic Score for CD (SES-CD).

Healthy controls (H) consisted of healthy volunteers and patients presenting with mild gastrointestinal symptoms in whom comprehensive diagnostic workups, including endoscopic examinations, laboratory analyses, and imaging studies, revealed no evidence of disease. All individuals were thoroughly evaluated to rule out IBD, infections, neoplasms, or other organic pathologies. Their symptoms were limited to nonspecific discomfort, such as functional dyspepsia or irregular bowel habits, without any objective pathological findings. Exclusion criteria were as follows: age < 18 years, inconclusive IBD diagnosis after follow-up, or IBD-unclassified. The respective results section indicates the number of subjects included.

We collected serum samples from 41 healthy individuals (controls), 86 patients with UC, and 68 patients with CD.

The patients were stratified into two clinical scenarios, “remission group” and “active disease group,” based on biochemical parameters (C-reactive protein/CRP and fecal calprotectin/FC levels) and disease-specific activity indices. Normal thresholds were defined as CRP < 5 mg/L and fecal calprotectin < 50 µg/g. For UC, clinical and endoscopic severity were assessed using the Mayo Clinic Score and Mayo Endoscopic Subscore, respectively. For CD, the Simple Endoscopic Score (SES) and the Harvey–Bradshaw index (HBI) were employed.

The remission group met all three criteria: normal CRP (<5 mg/L) and fecal calprotectin (<50 µg/g); minimal disease activity scores (Mayo Clinic Score ≤ 5 with no subscore > 1 for UC; HBI ≤ 4 for CD) and endoscopic remission (Mayo Endoscopic Subscore < 1 for UC; SES ≤ 2 for CD).

The medical records of the participants were reviewed to determine whether, after their enrollment in the study, any modifications or intensifications of their pharmacological treatment had been made, and whether any surgical interventions had been performed. This review was performed in order to assess potential relationships between the studied parameters and the prognosis of the pathologies. Those patients who needed intensification in their medication or needed IBD-related surgery were classified as bad prognosis.

Ethical approvals were obtained from the Central Norway Regional Committee for Medical and Health Research Ethics (reference numbers 5.2007.910 and 22687), and all patients gave informed written consent.

### 2.2. Study Variables

The study evaluated the following variables: (1) demographic and clinical characteristics: age, sex, smoking status, age at diagnosis, and disease duration; (2) disease classification: Montreal classification for UC and CD subtypes; (3) surgical history: prior surgical interventions; (4) clinical activity assessment: UC (Mayo Score and Mayo Endoscopic Score), CD (HBI and SES) and biomarkers: plasma CRP levels and FC concentrations [CRP: samples were analyzed using Human CRP ELH-CRP-5 (RayBiotech, Inc. Norcross, GA, USA). ELISA assays were executed according to the manufacturer’s protocol. FC: calprotectin in the same samples was analyzed using ELISA by Calpro AS, Lysaker, Norway. The samples were diluted 1:50 using Calpro EasyExtract and further diluted according to the manufacturer’s protocol]. Clinical status: stratification into remission or active disease phases. Pharmacological management: current treatments, including immunomodulators, biologics, and corticosteroids. Therapeutic adjustments: treatment modification/intensification or surgical interventions postenrollment. Immunological markers: anti-*A. simplex* antibodies (total immunoglobulins [Igs], IgG, IgM, IgA, IgE) and anti-*P. decipiens* antibodies (IgG, IgA, IgE).

### 2.3. Determination of Specific Antibodies

Third-stage larvae (L3) of *A. simplex* and *P. decipiens* were isolated from the musculature of *Micromesistius poutassou* (blue whiting) and *Lophius piscatorius* (monkfish), respectively. Larval specimens were homogenized following sequential sonication and PBS extraction, adhering to the methodological framework established by Perteguer and Cuéllar [30].

The ELISA methodology employed in this study utilized Costar microplates (Corning, NY, USA) coated with 10 μg/mL of total larval antigen. Human serum samples were diluted 1:100 in PBS–Tween buffer supplemented with 0.1% bovine serum albumin (BSA) and incubated to facilitate antigen–antibody binding. For immunoglobulin detection, horseradish peroxidase (HRP)-conjugated goat antihuman secondary antibodies (Biosource International, Camarillo, CA, USA) specific to Igs, IgM, IgG, or IgA were employed. IgE quantification required serum dilution at 1:2, followed by sequential incubations with a murine antihuman IgE monoclonal antibody (clone E21A11, IgG1Ƙ isotype; INGENASA, Madrid, Spain) and HRP-conjugated goat antimouse IgG1 antibodies (Life Technologies, Grand Island, NY, USA) [31,32].

To compare mean values of quantitative variables, participants were stratified into two groups based on their antibody titers against both studied anisakid species. Seropositivity was defined as optical density (OD) values exceeding the mean plus two standard deviations of the healthy control cohort.

### 2.4. Statistical Analysis

Normality assumptions were assessed using the Kolmogorov–Smirnov test. For quantitative variables meeting normality criteria, independent samples *t*-tests were applied to compare group means. When normality assumptions were violated, the nonparametric Mann–Whitney *U* test was employed. Correlational analyses were performed using Pearson’s coefficient for continuous variables in parametric populations, while Spearman’s rho coefficient was utilized for nonparametric datasets. Associations between categorical variables were evaluated through chi-square tests and Pearson’s correlation coefficient, as appropriate. Statistical significance was defined at *p* < 0.05, with a 95% confidence interval. All analyses were conducted using IBM SPSS Statistics, version 29.

## 3. Results

### 3.1. Population Data Summary

Table 1 shows the numbers and percentages of men and women, the number of smokers, the mean age, the number of individuals aged 18 to 39 years, and the number of individuals over 40 years of age in healthy controls, UC patients, and CD patients.

### 3.2. Patient Characteristics

Table 2 presents data on patients with UC and CD, detailing their Montreal classification phenotype, time from diagnosis to study inclusion and sample collection, age at diagnosis, and surgical history.

### 3.3. Antibody Values and Positivity Rates Between Populations

#### 3.3.1. Antibody Values

**Figure 1:**
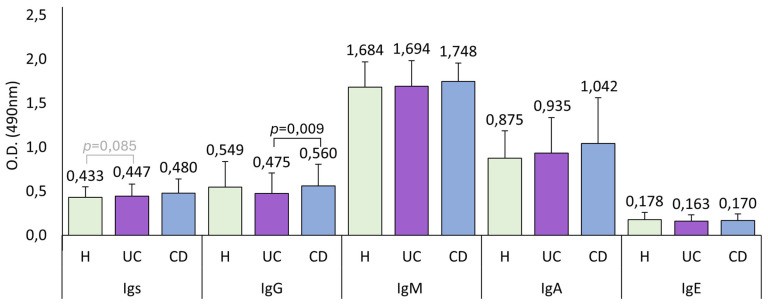
Levels of anti-*Anisakis simplex* antibodies were measured in serum samples from healthy controls (H, n = 41) and ulcerative colitis (UC, n = 86) and Crohn’s disease (CD, n = 68) patients. Data are presented as mean optical densities (O.D.) with error bars representing standard deviations.

**Figure A1:**
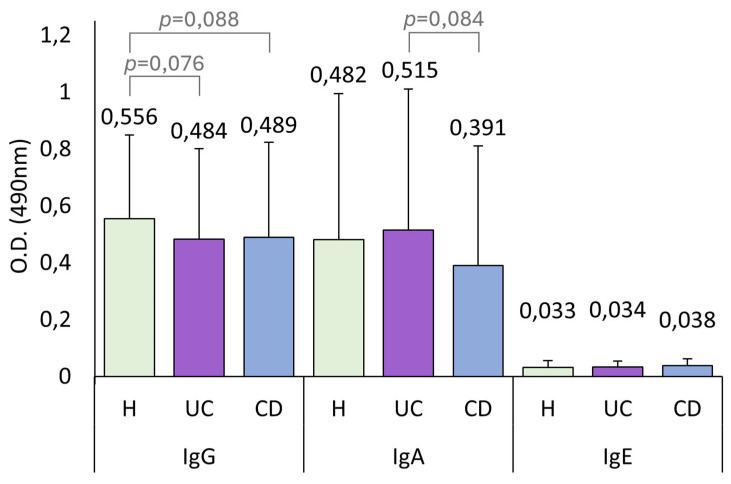
Levels of anti-*Pseudoterranova decipiens* antibodies were measured in serum samples from healthy controls (H, n = 41), ulcerative colitis patients (UC, n = 86), and Crohn’s disease (CD, n = 68) patients. Data are presented as mean optical densities (O.D.) with error bars representing standard deviations.

CD patients had significantly higher anti-*A. simplex* IgG levels than UC patients (*p* = 0.009). No significant differences were found between healthy controls and UC and CD patients. Figure 1 displays anti-*A. simplex* antibody levels across the three groups; Figure A1 shows anti-*P. decipiens* antibody levels.

#### 3.3.2. Antibody Positivity

**Figure 2:**
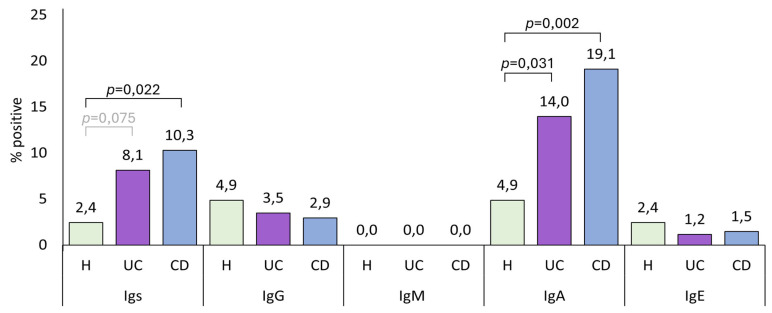
The percentages of anti-*Anisakis simplex* seropositivity (defined as optical density values exceeding the mean + 2 standard deviations) were analyzed in healthy controls (H; n = 41) and ulcerative colitis (UC; n = 86) and Crohn’s disease (CD; n = 68) cohorts.

**Figure A2:**
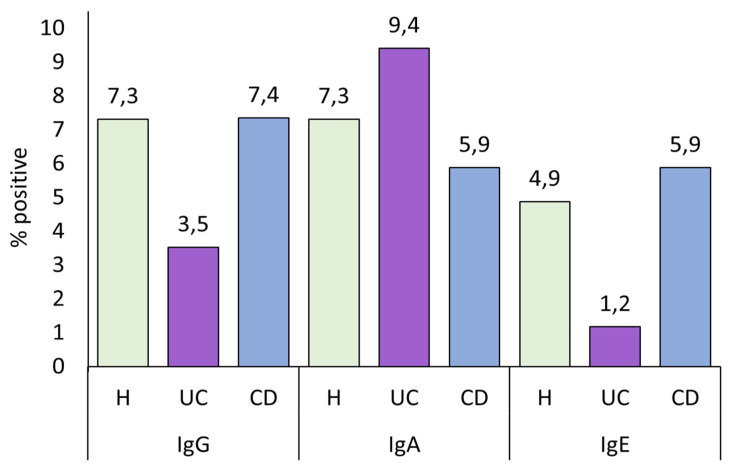
The percentages of anti-*Pseudoterranova decipiens* seropositivity (defined as optical density values exceeding the mean + 2 standard deviations) were analyzed in healthy controls (H; n = 41), ulcerative colitis patients (UC; n = 86), and Crohn’s disease (CD; n = 68) patients. Statistical comparisons between groups were performed using chi-square tests as appropriate for categorical data analysis.

The number of positive cases for each anti-*A. simplex* and anti-*P. decipiens* immunoglobulin was compared, defining positivity as optical density (O.D.) values greater than the mean plus two standard deviations of the healthy control group. CD patients were significantly more positive for anti-*A. simplex* IgA than healthy controls (*p* = 0.037), though IgA positivity did not increase the probability of developing CD (OR = 4.609 [0.984, 21.591]). Compared with healthy controls, the percentages for UC- and CD-positive patients were 3.4 and 4.3 times higher, respectively, for total anti-*A. simplex* immunoglobulins, and 2.9 and 3.9 times higher, respectively, for anti-*A. simplex* IgA. Figure 2 shows percentages of anti-*A. simplex* antibody positivity; Figure A2 presents anti-*P. decipiens* positivity rates. Table A1 details the number and percentage of positive cases of each anti-*A. simplex* and anti-*P. decipiens* antibody for healthy controls and UC and CD patients.

### 3.4. Antibody Levels and Demographic Characteristics

#### 3.4.1. Sex

In healthy controls, women exhibited significantly higher anti-*A. simplex* IgM levels than men (*p* = 0.005; men = 1.534 ± 0.254; women = 1.780 ± 0.268), while men had higher anti-*A. simplex* IgE levels than women (*p* = 0.003; men = 0.224 ± 0.095; women = 0.148 ± 0.058). Among CD patients, women showed higher total anti-*A. simplex* antibody levels than men (*p* = 0.049; men = 0.425 ± 0.130; women = 0.507 ± 0.167). Sex did not increase the probability of developing UC or CD, but women were more likely to develop CD than UC (*p* = 0.028; OR = 2.091 [1.080, 4.049]). Table A2 details anti-*A. simplex* and anti-*P. decipiens* antibody levels by sex across groups and presents the male-to-female ratios.

#### 3.4.2. Smoking Habits

UC patients who smoked had lower total specific antibody levels (*p* = 0.015; nonsmokers = 0.468 ± 0.133; smokers = 0.391 ± 0.121) and lower anti-*A. simplex* IgM (*p* = 0.002; nonsmokers = 1.752 ± 0.250; smokers = 1.542 ± 0.334). CD patients who smoked also had significantly lower anti-*A. simplex* IgM levels (*p* = 0.011; nonsmokers = 1.796 ± 0.189; smokers = 1.666 ± 0.214). No significant differences in antibody levels were observed in healthy controls based on smoking status. Smoking did not increase the likelihood of developing UC or CD. No differences were observed in anti-*P. decipiens*. Table A3 presents antibody levels by smoking status and the distribution of smokers across groups.

#### 3.4.3. Age Correlations

In healthy controls, age showed a significant inverse correlation with anti-*A. simplex* IgM (*p* < 0.001; Pearson’s r = −0.496). In UC patients, age inversely correlated with anti-*A. simplex* IgM (*p* < 0.001; Spearman’s rho = −0.354) and total immunoglobulins (*p* = 0.064; Pearson’s r = −0.201) but positively correlated with anti-*P. decipiens* IgA (*p* = 0.001; Spearman’s rho = 0.341) and IgG (*p* = 0.058; Spearman’s rho = 0.206). In CD patients, age inversely correlated with anti-*A. simplex* IgE (*p* = 0.024; Spearman’s rho = −0.273) and IgM (*p* = 0.086; Spearman’s rho = −0.210) and positively with anti-*A. simplex* IgA (*p* = 0.055; Spearman’s rho = 0.234). No differences were observed in anti-*P. decipiens*. Table A4 provides correlation coefficients for antibody levels and age in all groups.

### 3.5. Antibody Levels and Phenotype

#### 3.5.1. Antibody Levels by Montreal Classification

CD patients with a colonic phenotype exhibited significantly higher anti-*P. decipiens* IgG levels (mean = 0.641 ± 0.361) than those with ileal (*p* = 0.019; mean = 0.331 ± 0.179) and ileocolonic (*p* = 0.049; mean = 0.471 ± 0.368) phenotypes. Table A5 provides detailed anti-*A. simplex* and anti-*P. decipiens* antibody levels according to the Montreal classification phenotype for both UC and CD patients.

#### 3.5.2. Correlation Between Antibody Levels and Disease Duration

In UC patients, positive correlations were found between time from diagnosis to study enrollment and the following antibody levels: Anti-*A. simplex* IgG (*p* = 0.017; Spearman’s rho = 0.258), anti-*A. simplex* IgE (*p* = 0.009; Spearman’s rho = 0.283), and anti-*P. decipiens* IgA (*p* = 0.007; Spearman’s rho = 0.294). In CD patients, anti-*A. simplex* IgA showed a positive correlation (*p* < 0.001; Spearman’s rho = 0.403), while anti-*A. simplex* IgE showed a negative trend (*p* = 0.064; Spearman’s rho = −0.223). Table A6 provides the full set of correlation coefficients between antibody levels and Montreal classification phenotypes.

#### 3.5.3. Impact of Surgery on Antibody Levels

Only CD patients underwent surgery; no UC patients had surgical intervention. CD patients with a history of surgery had higher levels of anti-*A. simplex* IgA (*p* = 0.037; without surgery = 0.952 ± 0.521; with surgery = 1.188 ± 0.495), and anti-*P. decipiens* IgA (*p* = 0.057; without surgery = 0.325 ± 0.347; with surgery = 0.499 ± 0.507). Table A7 details antibody levels in CD patients by surgical history.

#### 3.5.4. Association of Smoking with Surgery and Antibody Levels

Smoking was significantly associated with an increased likelihood of requiring surgery (*p* = 0.005; OR = 4.364 [1.522, 12.511]). However, smoking did not correlate with higher anti-*A. simplex* IgA levels.

### 3.6. Antibody Levels and Clinical Activity

#### 3.6.1. Antibody Levels in Healthy Control vs. Ulcerative Colitis and Crohn’s Disease in Remission and Active Patients

**Figure 3:**
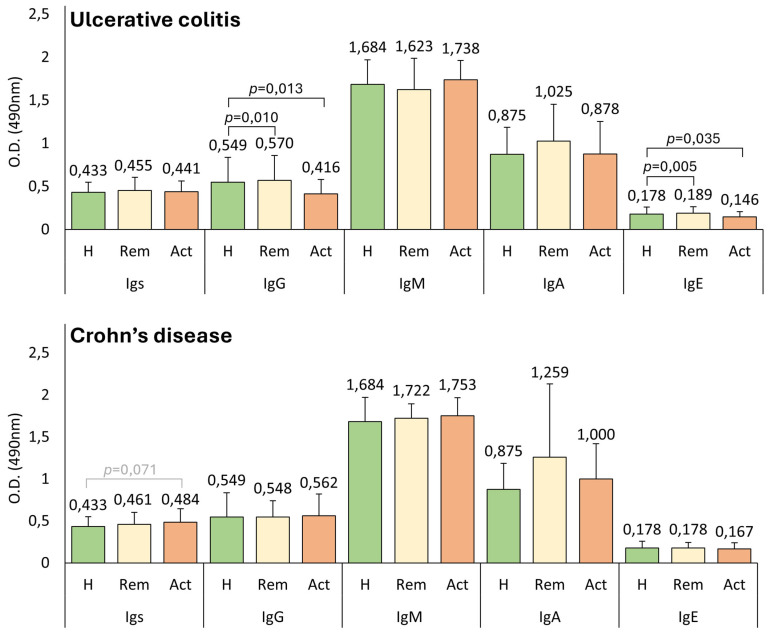
Comparative analysis of anti-*Anisakis simplex* antibody levels in ulcerative colitis and Crohn’s disease: Associations with disease activity. Serum anti-*Anisakis simplex* antibody levels were evaluated in ulcerative colitis (UC, n = 86), Crohn’s disease (CD, n = 68), and healthy controls (H, n = 41). UC patients were stratified into remission (Rem; n = 33, 38.4%) and active disease (Act; n = 53, 61.6%) groups, and CD patients were categorized into remission (Rem; n = 11, 16.2%) and active disease (Act; n = 57, 82.8%) groups. Antibody levels were expressed as mean optical densities (O.D.) with standard deviations (T-bars).

**Figure A3:**
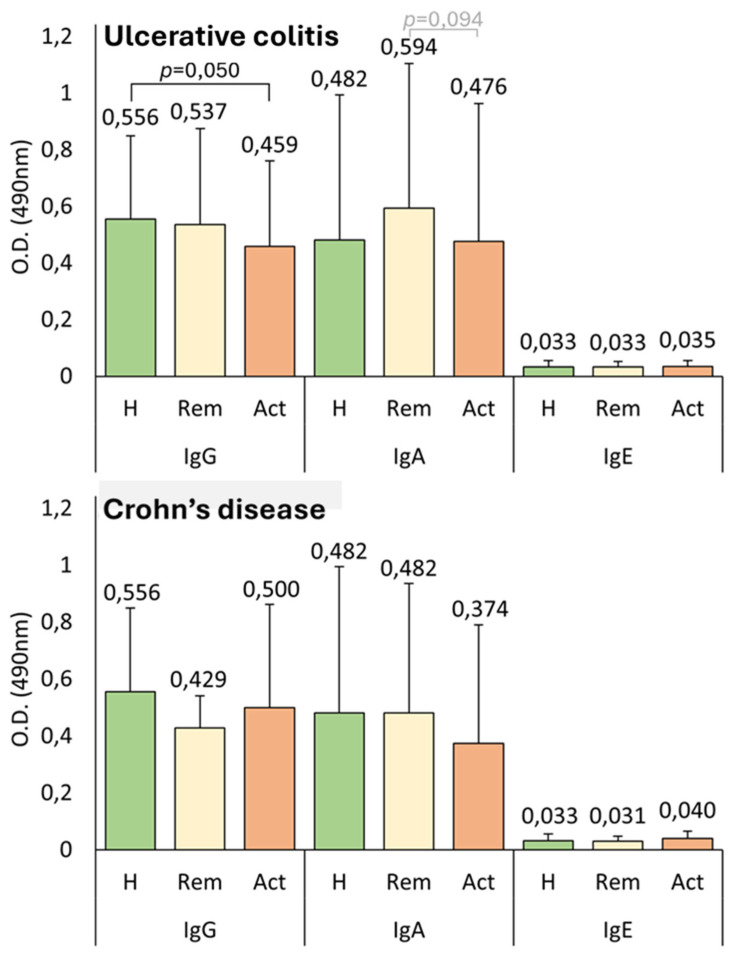
Comparative analysis of anti-*Pseudoterranova decipiens* antibody levels in ulcerative colitis and Crohn’s disease: associations with disease activity. Serum anti-*P. decipiens* antibody levels were evaluated in ulcerative colitis patients (UC, n = 86), Crohn’s disease patients (CD, n = 68), and healthy controls (H, n = 41). UC patients were stratified into remission (Rem; n = 33, 38.4%) and active disease (Act; n = 53, 61.6%) groups, and CD patients were categorized into remission (Rem; n = 11, 16.2%) and active disease (Act; n = 57, 82.8%) groups. Antibody levels are expressed as mean optical densities (O.D.) with standard deviations (T-bars).

Anti-*A. simplex* and anti-*P. decipiens* antibody levels were measured in UC and CD patients, stratified by clinical activity (active vs. remission), and compared with healthy controls. Healthy controls and UC patients in remission exhibited significantly higher levels of anti-*A. simplex* IgG and IgE than clinically active UC patients. Conversely, healthy controls had significantly higher levels of anti-*P. decipiens* IgG than active UC patients. Figure 3 provides means and standard deviations of the anti-*A. simplex* antibody levels of active and in-remission UC and CD patients compared with those of healthy controls. Figure A3 provides means and standard deviations of anti-*P. decipiens* antibody levels.

#### 3.6.2. Antibody Levels and Activity Scales

Table 3 provides the numbers and percentages of UC and CD active and in-remission patients according to clinical scales, mean values of clinical scale valuations, and the numbers and percentages of UC and CD patients with good prognosis.

#### 3.6.3. Correlation with Activity Scales

Significant inverse correlations were observed in UC patients between the Mayo Endoscopic Score and both anti-*A. simplex* IgG (*p* = 0.002; Spearman’s rho = −0.328) and IgE (*p* < 0.001; Spearman’s rho = −0.397). Similar negative correlations were seen between the Mayo Score and anti-*A. simplex* IgG (*p* = 0.013; Spearman’s rho = −0.272) and IgE (*p* < 0.001; Spearman’s rho = −0.361). These findings indicate that higher disease activity could be associated with lower IgG and IgE anti-*A. simplex* antibody levels. Table A9 shows the correlations between anti-*A. simplex* and anti-*P. decipiens* antibodies and Activity Scale valuations.

#### 3.6.4. Association with Inflammatory Markers (C-Reactive Protein and Calprotectin)

UC patients with normal CRP levels had higher anti-*A. simplex* IgA (*p* = 0.017; normal = 0.965 ± 0.403; elevated = 0.719 ± 0.287) and anti-*P. decipiens* IgG (*p* = 0.026; normal = 0.553 ± 0.352; elevated = 0.356 ± 0.231) than those with elevated CRP. Significant inverse correlations were found in UC patients between CRP and anti-*A. simplex* IgA (Spearman’s rho −0.308, *p* = 0.015) and anti-*P. decipiens* IgG (Spearman’s rho −0.256, *p* = 0.046). In CD patients, calprotectin levels were inversely correlated with anti-*A. simplex* IgM (Spearman’s rho −0.298, *p* = 0.050). Table 4 provides mean values of CRP and calprotectin of UC and CD patients. Table A10 provides means and standard deviations for anti-*A. simplex* and anti-*P. decipiens* antibodies in UC and CD’s patients stratified by CPR and calprotectin levels. Table A11 provides the correlation between anti-*A. simplex* and anti-*P. decipiens* antibodies and CPR and calprotectin levels.

### 3.7. Antibody Levels and Prognosis

Prognosis was evaluated based on the need for additional or intensified treatment and surgical intervention. Patients were considered to have bad prognosis when either treatment intensification, surgical procedures related to IBD, or both were needed during follow-up. UC patients with good prognoses had higher levels of anti-*A. simplex* IgG, IgE, and anti-*P. decipiens* IgG (*p* = 0.004; good = 0.549 ± 0.324; poor = 0.383 ± 0.288) than those with poor prognoses. CD patients with good prognoses had significantly higher anti-*A. simplex* IgM. In treatment-based prognosis, UC patients with good prognoses had higher anti-*A. simplex* IgG and anti-*P. decipiens* IgG (*p* = 0.001; good = 0.551 ± 0.326; poor = 0.351 ± 0.262), while CD patients with good prognoses had higher anti-*A. simplex* IgM. No significant differences in antibody levels were observed based on bad prognoses based on the need for IBD-related surgical intervention. Table 5 provides means and standard deviations of *A. simplex* antibody levels in UC and CD patients stratified by prognosis based on the need of medical treatment escalation. Table A12 provides means and standard deviations of *A. simplex* antibody levels in UC and CD patients stratified by prognosis based on the need for IBD-related surgical intervention and *P. decipiens* antibody levels in UC and CD patients stratified by prognosis based on the need for medical treatment escalation and prognosis based on the need for IBD-related surgical intervention.

### 3.8. Antibody Levels and Treatment

The relationship between anti-*A. simplex* and anti-*P. decipiens* antibody levels in patients with UC and CD was analyzed according to the immunosuppressive treatments administered at the time of inclusion in the study.

UC patients receiving immunosuppressive therapy, including corticosteroids, azathioprine, and anti-TNF-α agents, demonstrated significantly lower levels of anti-*A. simplex* IgG (*p* = 0.010; without immunosuppressants = 0.518 ± 0.247; with immunosuppressants = 0.383 ± 0.172) and IgE (*p* = 0.036; without immunosuppressants = 0.173 ± 0.067; with immunosuppressants = 0.140 ± 0.071) than those who had not received immunosuppressants. When corticosteroid treatment was considered individually, UC patients treated with corticosteroids exhibited lower anti-*A. simplex* IgG (*p* = 0.005; without corticosteroids = 0.508 ± 0.240; with corticosteroids = 0.360 ± 0.169) and IgE levels (*p* = 0.023; without corticosteroids = 0.172 ± 0.066; with corticosteroids = 0.131 ± 0.075).

Conversely, UC patients treated with azathioprine (*p* = 0.042; without azathioprine = 0.911 ± 0.395; with azathioprine = 1.249 ± 0.408) or anti-TNF-α agents (*p* = 0.021; without anti-TNF-α= 0.908 ± 0.392; with anti-TNF-α= 1.294 ± 0.413) had significantly higher levels of anti-*A. simplex* IgA.

Furthermore, UC patients treated with anti-TNF-α agents exhibited higher anti-*A. simplex* IgM levels than both untreated UC patients (*p* = 0.036; without anti-TNF-α = 1.677 ± 0.291; with anti-TNF-α = 1.911 ± 0.179) and CD patients (*p* = 0.004; without anti-TNF-α = 1.725 ± 0.207; with anti-TNF-α = 1.919 ± 0.110). Additionally, UC patients receiving anti-TNF-α therapy showed increased anti-*P. decipiens* IgE levels (*p* = 0.042; without anti-TNF-α = 0.033 ± 0.019; with anti-TNF-α = 0.050 ± 0.050).

Table A13 shows the numbers and percentages of CU and CD patients treated with any treatment and the data for each treatment.

## 4. Discussion

The present work investigates the seroprevalence of antibodies against *A. simplex* in Norwegian patients with IBD, specifically UC and CD, and compares these findings with those in healthy controls. The research also explores associations between anti-*A. simplex* antibody positivity and various clinical and laboratory parameters in IBD.

This study provides novel insights into the immunological landscape of IBD patients in Norway regarding exposure to *A. simplex*, a nematode commonly acquired through fish consumption. There are few data on the prevalence of *A. simplex* infection in Nordic European countries, with sensitization rates close to 0%, despite reports of a high prevalence of anisakids in fish intended for human consumption [33,34,35,36,37,38,39,40].

The values of *A. simplex* seroprevalence in Norway, which is considered one of the countries with the highest fish consumption, were very low. Therefore, we also studied the seroprevalence of *P. decipiens* to determine whether these findings could be due to Norwegian fish being preferentially parasitized by this other anisakid. The results obtained for *P. decipiens* were not substantially different, and we found similarities with the results obtained with *A. simplex*. Therefore, we consider that the results obtained with *P. decipiens* complement and reinforce the results obtained with *A. simplex*.

IBD, including UC and CD, is a chronic inflammatory condition of the gastrointestinal tract with a stable incidence in Norway [1,2]. The etiopathogenesis of IBD is multifactorial, involving genetics, environment, flora, and immune response, with lifestyle changes in industrialized countries potentially contributing through altered immune system development [6].

*A. simplex*, a nematode parasite, can cause infections that resemble CD, and previous studies have shown a high prevalence of anti-*A. simplex* antibodies in IBD patients, which may be linked to alterations in innate immunity [16,20,21].

Several studies have observed a relationship between serum immunoglobulin levels and CD phenotype [41,42,43,44,45,46,47]. Interestingly, other studies have described the usefulness of antibodies against certain microorganisms as possible markers of these diseases. The potential use of ASCA for CD and ANCA for UC as markers for characterizing the pathologies has been studied, since these antibodies are elevated before the appearance of the first symptoms and sings of the disease [48,49,50,51,52]. Therefore, we have investigated whether antibodies against *A. simplex* could be useful in the diagnosis and/or monitoring of these diseases.

The population sizes in our study were selected based on previous articles concerning *Anisakis* seroprevalence and prevalence in Norway [33,34]. Patient selection was not consciously made based on sex (given the female preponderance in immune-mediated diseases) [48] or smoking habits [49], despite these being important factors in these conditions.

UC patients had lower IgG levels than both healthy controls and CD patients, although this difference is statistically significant only when compared with CD patients. As we discuss later, lower IgG levels are linked to a worse disease state. Therefore, having low IgG levels could be a biomarker of the development of the pathology. In the present study, IBD patients had higher total anti-*A. simplex* Igs and IgA seroprevalence than healthy controls. Although not statistically significant, we observed the same pattern in these immunoglobulin levels, where CD patients exhibited higher levels than UC patients, and both groups had higher levels than healthy controls, as shown in Figure 1.

In a study we conducted in Spain, the seroprevalence of IgA anti-*A. simplex* in the healthy population was higher than in this study conducted in Norwegian individuals [50]. This observation is further supported by the variations in fish consumption habits among the inhabitants of these countries [51]. IgA antibodies are produced upon ingestion of the parasite’s protein, even if the larvae are dead. In contrast, IgE antibodies are generated only when a live larva is ingested [50,52].

Women in the healthy control group had higher anti-*A. simplex* IgM levels, while men had higher IgE levels. Among CD patients, women exhibited higher total anti-*A. simplex* antibody levels than men. The sex-specific differences in antibody levels, particularly the higher total antibody titers in CD women, may reflect underlying immunological or behavioral factors, such as dietary habits or hormonal influences on immune function.

In our study, smoking was associated with lower IgM anti-*A. simplex* antibody levels in both UC and CD patients. This finding aligns with known immunomodulatory effects of tobacco, but its clinical significance in the context of parasitic exposure and IBD pathogenesis warrants further investigation. Smoking has distinct immunological and clinical effects in UC and CD. Other studies have linked tobacco consumption in UC to better disease state likely due to its anti-inflammatory activity in this condition [49]. However, this protective effect is counterbalanced by an increased risk of cancer and overall mortality in smokers [53,54]. In contrast, in CD, smoking is linked to a worsened disease course, with higher rates of complications and surgical interventions [53,55]. In our study, we observed a relationship in CD where smoking increased the likelihood of surgical intervention. Thus, while smoking lowers certain antibody levels in both UC and CD, it confers some kind of protection only in UC, whereas in CD, it exacerbates disease severity and adverse outcomes.

Age showed an inverse correlation with anti-*A. simplex* IgM in healthy controls and UC and CD patients. In CD, age correlated inversely with IgE and IgM, but positively with IgA. The inverse relationship between age and certain antibody isotypes (notably IgM) could indicate waning exposure or immune responsiveness with age, or possibly immunosenescence.

In patients with UC, we observed positive correlations between disease duration (defined as the time from diagnosis to study inclusion) and serum levels of both IgG and IgE antibodies against *A. simplex*. In contrast, among patients with CD, disease duration was positively correlated with IgA levels and negatively correlated with IgE levels. It is important to note that serum IgA should not be equated with mucosal secretory IgA, although elevated serum IgA may reflect systemic antigenic exposure, particularly in individuals with increased intestinal permeability. Additionally, IgE antibodies to *Anisakis* can persist even after clearance of infection, and sensitization does not always indicate current active infection. We found no significant results regarding the relationship among disease phenotype, extent of pathology, and anti-*A. simplex* levels. While other studies have linked the extent of affected intestine to disease severity and prognosis [56], our findings suggest that a larger area of intestinal damage does not increase the immune response to *A. simplex*.

Surgically treated CD patients exhibited higher levels of IgA anti-*A. simplex*. This relationship is challenging to interpret with the current results, but it is plausible that elevated IgA levels could serve as a marker for CD development, since that IgA is higher in CD patients than in healthy controls, as previously discussed. We also observed that, in UC, lower levels of anti-*A. simplex* IgG, along with IgE, were associated with higher clinical activity, worse colonic condition according to the Mayo Endoscopic Score, and worse prognosis. Notably, none of these relationships with any immunoglobulin were observed in CD. Higher levels of anti-*A. simplex* IgG and IgE in UC associated with less severe disease could indicate that *A. simplex* downregulates the inflammatory response in ulcerative colitis [26,28].

It’s also interesting to observe that as the disease progressed over time, CD patients exhibited decreased IgE levels, while UC patients showed increased IgE levels. Furthermore, as we found in this study, higher IgE levels in UC correlated with a better disease state.

These results may suggest a differential immune response or exposure pattern between these IBD subtypes. This could result from a combination of broader immune reactivity and anatomical differences (more frequent small-intestinal and transmural involvement). CD is characterized by a heightened immune response to a wide range of microbial and food antigens, reflected in elevated serum IgG reactivity to multiple antigens. CD often affects the small intestine and involves transmural (full thickness) inflammation, whereas UC is restricted to the colonic mucosa. The small intestine contains more lymphoid aggregates, which can promote greater antibody production against luminal antigens such as *A. simplex* [57,58,59,60].

Another interesting finding is that patients with UC and CD in remission exhibited higher IgA levels than active patients and healthy controls, as illustrated in Figure 3. The lack of statistical significance in the test could be attributed to the sample sizes generated when analyzed by groups. This observation allows for the hypothesis that while damaged intestinal mucosa and altered permeability might facilitate the access of *Anisakis* proteins to Peyer’s patches, patients in remission are immunocompetent and thus possess a greater capacity to react to this increased antigenic exposure. The group of patients with CD in remission (n = 11) was notably small, primarily because of the inherent challenges of recruiting individuals who had recently undergone endoscopy, exhibited normal biomarker levels, and maintained stable therapy at the time of study inclusion. Consequently, we refrain from drawing definitive conclusions for this group, and the findings observed in this subgroup should be interpreted as nongeneralizable trends. In UC patients, those with normal C-reactive protein (CRP) levels also exhibited higher IgA levels, and there was also an inverse correlation observed between CRP and IgA values. Other studies have demonstrated an immunomodulatory role for IgA in various immune-mediated diseases, where elevated IgA levels are associated with an improved inflammatory state [61].

Immunosuppressive therapy, particularly with corticosteroids, was associated with reduced anti-*A. simplex* IgG and IgE responses in UC patients, while azathioprine and anti-TNF-α treatments were linked to elevated anti-*A. simplex* IgA, and anti-TNF-α therapy was associated with increased anti-*A. simplex* IgM and anti-*P. decipiens* IgE levels. Furthermore, both UC and CD patients treated with anti-TNF-α agents exhibited higher anti-*A. simplex* IgM levels than untreated IBD patients. Higher anti-*A. simplex* IgM levels in IBD patients receiving anti-TNF-α therapy may reflect an altered or enhanced early humoral immune response to *A. simplex* antigens likely due to the immunomodulatory effects of anti-TNF-α treatment on B-cell function and mucosal immunity. While anti-TNF-α agents suppress inflammation by blocking TNF-α, they can also induce broader changes in immune regulation, including shifts in antibody production and the induction of various autoantibodies [62]. The observed increase in IgM suggests that anti-TNF-α therapy does not uniformly suppress all antibody responses and may selectively influence the production of certain isotypes, such as IgM, possibly as a compensatory or adaptive mechanism. The clinical implications of this finding are not yet fully understood, but it highlights the need for further research into how anti-TNF-α therapy affects immune responses to parasitic antigens and potential infection risks in treated patients [62,63].

The study’s results support the hypothesis that altered mucosal immunity in IBD may facilitate sensitization to *A. simplex* antigens. This is consistent with previous reports of increased antiparasite antibody prevalence in IBD and suggests that immune dysregulation in these patients extends to responses against environmental antigens [64,65]. The detected antibodies may reflect a historical immune response, potentially cross-reactive or facilitated by the dysbiosis and increased intestinal permeability characteristic of IBD, rather than indicating an active parasitic infection. Similarly, serological testing represents a valid epidemiological tool for assessing immune sensitization. However, the presence of these antibodies does not distinguish between past exposure, cross-reactivity, or other mechanisms of immune activation. Other studies have described the usefulness of antibodies against certain microorganisms as possible markers of IBD. Therefore, we investigated whether antibodies against *A. simplex* could be useful in the diagnosis and/or monitoring of these diseases. Elevated antimicrobial antibody levels are robustly correlated with more severe, complicated, and aggressive forms of CD, and to a lesser extent, with severe UC. These serological markers can help predict disease progression, need for surgery, and risk of complications in both conditions [54,66,67].

We were mindful of the inferential limitations of our study and have presented our findings as preliminary associations that require future validation before antibodies can be definitively proposed as clinical biomarkers. Our research should be regarded as exploratory and hypothesis-generating rather than confirmatory. The robustness and clinical relevance of the study could be enhanced through several methodological and analytical improvements. For instance, a longitudinal design would allow for the assessment of temporal relationships and potential causality between anti-*A. simplex* antibody levels and disease activity, as well as the predictive value of these biomarkers. Adjusting for confounding variables such as dietary habits and consumption of raw or undercooked fish would improve the specificity of the observed associations, given the geographic, occupational, and cultural variability in the risk of exposure to marine helminths. In our cohort, these data were not systematically collected, which precluded rigorous multivariate analysis. Thus, future longitudinal and mechanistic studies are needed to clarify the predictive or pathogenic significance of these serological responses. We also recommend comprehensive assessment of participants’ dietary habits as a priority in future longitudinal or multicenter studies, in addition to genetic profiling and characterization of the gut microbiota. Further experimental evidence is needed to establish the functional or biomarker implications of anti-*Anisakis* antibodies, and longitudinal validation is necessary to confirm the association between serological findings and clinical outcomes.

## 5. Conclusions

This study provides compelling evidence that patients with IBD, specifically UC and CD, exhibit distinct serological profiles of antibodies against the parasitic nematode *A. simplex* and related parasites. Notably in UC, higher levels of anti-*A. simplex* IgG and IgE were associated with clinical remission, suggesting a potential protective or modulatory role of these antibodies in disease activity. Furthermore, the research reveals that immunosuppressive treatments influence antibody levels, with corticosteroids decreasing and azathioprine and anti-TNF-α agents increasing specific antibody responses. These findings support the hypothesis that immune responses to *A. simplex* may be intricately linked to IBD pathogenesis, disease prognosis, and therapeutic response, highlighting the potential utility of parasite-specific antibodies as biomarkers for disease monitoring and understanding host–parasite interactions within the gut immune environment.

## Figures and Tables

**Table 1:** Demographics and clinical characteristics of healthy controls, ulcerative colitis patients, and Crohn’s disease patients: sex distribution, smoking status, mean age, and age stratification (18–39 vs. ≥40 years).

Characteristic	Healthy (n = 41)	Ulcerative Colitis (n = 86)	Crohn’s Disease (n = 68)
**Sex**			
Men	16 (39.0%)	43 (50.0%)	22 (32.4%)
Women	25 (61.0%)	43 (50.0%)	46 (67.6%)
**Smoking status**	13 (31.7%)	24 (27.9%)	25 (36.8%)
**Age (years)**			
Mean ± SD	47.71 ± 14.94	38.85 ± 15.04	35.06 ± 12.73
18–39 years	11 (26.8%)	49 (57.0%)	49 (72.1%)
≥40 years	30 (73.2%)	37 (43.0%)	19 (27.9%)

**Table 2:** Clinical and demographic characteristics of inflammatory bowel disease patients.

Parameter	Ulcerative Colitis (n = 86)	Crohn’s Disease (n = 68)
**Montreal classification**		
- Proctitis (E1)	23 (27.7%)	-
- Left-sided colitis (E2)	41 (49.4%)	-
- Extensive colitis (E3)	19 (22.9%)	-
- Ileal (L1)	-	9 (15.5%)
- Colonic (L2)	-	19 (32.8%)
- Ileocolonic (L3)	-	30 (51.7%)
- Perianal involvement (P)	-	4 (6.9%)
**Disease duration (months)**		
Mean ± SD	121.56 ± 151.16	119.63 ± 110.86
- <3 months	12 (14.1%)	2 (2.9%)
- 3–120 months	41 (48.2%)	39 (57.4%)
- >120 months	32 (37.6%)	27 (39.7%)
**Age at diagnosis (years)**		
Mean ± SD	28.89 ± 12.79	25.12 ± 10.04
- <17 years	10 (8.6%)	14 (9.5%)
- 18–39 years	60 (51.6%)	49 (33.3%)
- ≥40 years	16 (13.8%)	5 (3.4%)
**Surgery**	0 (0%)	26 (38.2%)

**Table 3:** Clinical outcomes comparison: ulcerative colitis vs. Crohn’s disease.

Parameter	Ulcerative Colitis (n = 86)	Crohn’s Disease (n = 68)
**Remission Status**		
Remission	33 (38.4%)	11 (16.2%)
Active Disease	53 (61.6%)	57 (83.8%)
**Endoscopic Scores**		
Mayo Endoscopic Score	1.07 ± 1.10	–
Active Patients (Mayo > 0)	48 (58.5%)	–
SES-CD	–	5.00 ± 5.391
Active Patients (SES-CD > 2)	–	41 (60.3%)
**Disease Activity Scores**		
Mayo Clinical Score	3.75 ± 3.83	–
Active Patients (Mayo > 5)	64 (77.1%)	–
HB Index	–	5.13 ± 5.19
Active Patients (HB > 4)	–	29 (42.6%)
**Prognostic Indicators**		
Good Global Prognosis	51 (64.6%)	23 (37.1%)
Favorable Treatment Response	55 (69.6%)	27 (44.3%)
Surgical Avoidance	70 (88.6%)	49 (80.3%)

Data presented as mean ± standard deviation for continuous variables and n (%) for categorical variables. SES-CD: Simple Endoscopic Score for Crohn’s Disease; HB: Harvey–Bradshaw index. UC patients in remission (per Mayo Endoscopic Score) had significantly higher anti-*A. simplex* IgG (*p* = 0.020; remission = 0.554 ± 0.319; active = 0.412 ± 0.207) and IgE (*p* = 0.005; remission = 0.188 ± 0.076; active = 0.144 ± 0.068) than those with active disease. No significant differences were observed in CD patients. Table A8 provides detailed means and standard deviations of anti-*A. simplex* and anti-*P. decipiens* antibody levels stratified by the clinical status according to de Activity Scales.

**Table 4:** Comparative analysis of inflammatory biomarkers in colitis vs. Crohn’s disease.

Parameter	Colitis	Crohn’s Disease
**C-Reactive Protein**		
Mean ± SD (mg/L)	12.75 ± 27.24	15.60 ± 26.80
Elevated > 5 mg/L	18/62 (29%)	25/60 (41.7%)
**Calprotectin**		
Mean ± SD (μg/g)	1636.11 ± 1126.88	1028.57 ± 919.80
Elevated > 50 μg/g	16/17 (94.1%)	39/44 (88.6%)

**Table 5:** Means and standard deviations of anti-Anisakis simplex antibody optical densities in ulcerative colitis and Crohn’s disease patients stratified by prognosis (**A**) and treatment-based prognosis (**B**).

(**A**)						
**Parameter.**	**Ulcerative Colitis**			**Crohn’s Disease**		
	**Good (n = 51)**	**Poor (n = 28)**	***p*-Value**	**Good (n = 23)**	**Poor (n = 39)**	***p*-Value**
**Igs**	0.458 ± 0.147	0.418 ± 0.121	NS	0.495 ± 0.113	0.480 ± 0.181	NS
**IgG**	0.536 ± 0.265	0.387 ± 0.142	0.016	0.517 ± 0.193	0.592 ± 0.283	NS
**IgM**	1.667 ± 0.326	1.721 ± 0.247	NS	1.848 ± 0.122	1.703 ± 0.216	0.004
**IgA**	0.945 ± 0.391	0.868 ± 0.405	NS	1.081 ± 0.491	1.016 ± 0.453	NS
**IgE**	0.177 ± 0.070	0.144 ± 0.064	0.043	0.158 ± 0.067	0.175 ± 0.080	NS
(**B**)						
**Parameter**	**Ulcerative Colitis**			**Crohn’s Disease**		
	**Good (n = 55)**	**Poor (n = 24)**	***p*-Value**	**Good (n = 22)**	**Poor (n = 46)**	***p*-Value**
**Igs**	0.462 ± 0.148	0.401 ± 0.104	0.072	0.510 ± 0.113	0.469 ± 0.187	NS
**IgG**	0.530 ± 0.264	0.379 ± 0.122	0.016	0.539 ± 0.247	0.585 ± 0.265	NS
**IgM**	1.686 ± 0.324	1.686 ± 0.242	NS	1.854 ± 0.128	1.679 ± 0.214	0.004
**IgA**	0.968 ± 0.411	0.804 ± 0.338	0.097	1.083 ± 0.465	0.990 ± 0.464	NS
**IgE**	0.173 ± 0.069	0.147 ± 0.068	0.117	0.161 ± 0.064	0.175 ± 0.084	NS

NS = not statistically significant (*p* > 0.05). Values expressed as mean ± standard deviation.

## Data Availability

The original contributions presented in this study are included in the article. Further inquiries can be directed to the corresponding authors.

## Appendix A

**Table A1:** Numbers, percentages, ratios, and significance of anti-*Anisakis simplex* (**A**) and anti-*Pseudoterranova decipiens* (**B**) immunoglobulin-positive healthy controls (H), ulcerative colitis patients (UC), Crohn’s disease patients (CD), and UC and CD together as inflammatory bowel disease patients (IBD).

(**A**) *Anisakis simplex*																
		**Igs**			**IgG**			**IgM**			**IgA**			**IgM**		
	**N**	**n**	**%**	**IC95%**	**n**	**%**	**IC95%**	**n**	**%**	**IC95%**	**n**	**%**	**IC95%**	**n**	**%**	**IC95%**
H	41	1	2.4	−2.3, 7.1	2	4.9	−1.7, 11.5	0	0	0.0, 0.0	2	4.9	−1.7, 11,5	1	2.4	−2.3, 7.1
IBD	154	14	9.1	4.6, 13.6	5	3.2	0.4, 6.0	0	0	0.0, 0.0	25	16.2	10.4, 22.0	2	1.3	−0.5, 3.1
UC	86	7	8.1	2.3, 13.9	3	3.5	−0.4, 7,4	0	0	0.0, 0.0	12	14	6.7, 21.3	1	1.2	−1.1, 3.5
CD	68	7	10.3	3.1, 17.5	2	2.9	−1.1, 6.9	0	0	0.0, 0.0	13	19.1	9.8, 28.4	1	1.5	−1.4, 4.4
			ratio			ratio						ratio			ratio	
%IBD/%H			3.8			0.7						3.3			0.5	
%UC/%H			3.4			0.7						2.9			0.5	
%CD/%H			4.3			0.6						3.9			0.6	
		*p*	*p*		*p*	*p*					*p*	*p*		*p*	*p*	
H vs. IBD		0.156	0.042		0.618	0.542					0.061	0.009		0.598	0.564	
H vs. UC		0.216	0.075		0.707	0.595					0.127	0.031		0.589	0.483	
H vs. CD		0.128	0.022		0.602	0.465					0.037	0.002		0.715	0.645	
(**B**) *Pseudoterranova decipiens*																
		**IgG**			**IgA**			**IgE**								
	**N**	**n**	**%**	**IC95%**	**n**	**%**	**IC95%**	**n**	**%**	**IC95%**						
H	41	3	7.3	−0.7, 15.3	12	7.8	3.6, 12.0	2	4.9	−1.7, 11.5						
IBD	153	8	5.2	1.7, 8.7	8	9.4	3.2, 15.6	5	3.4	0.5, 6.3						
UC	85	3	3.5	−0.4, 7.4	4	5.9	0.3, 11.5	1	1.2	−1.1, 3.5						
CD	68	5	7.4	1.2, 13.6	3	7.3	−0.7, 15.3	4	5.9	0.3, 11.5						
			ratio			ratio			ratio							
%IBD/%H			0.7			1.1			0.7							
%UC/%H			0.5			1.3			0.2							
%CD/%H			1			0.8			1.2							
		*p*	*p*		*p*	*p*		*p*	*p*							
H vs. IBD		0.608	0.54		0.636	0.894		0.624	0.595							
H vs. UC		0.35	0.234		0.696	0.128		0.202	0.128							
H vs. CD		0.994	0.978		0.767	0.754		0.824	0.754							

N: number of participants in each group. n: number of positive people in each group. IC95%: confidence interval of the percentage of positive people.

**Table A2:** Mean optical density values and standard deviations for anti-*Anisakis simplex* (**A**) and anti-*Pseudoterranova decipiens* (**B**) antibody levels stratified by sex in healthy controls, ulcerative colitis patients, and Crohn’s disease patients. Sex distribution across study cohorts (**C**).

(**A**) *Anisakis simplex*												
	**Healthy**				**Ulcerative Colitis**				**Crohn’s Disease**			
	**Men**	**Women**			**Men**	**Women**			**Men**	**Women**		
	**16**	**25**		***p***	**43**	**43**		***p***	**22**	**46**		***p***
Igs	0.435 ± 0.153	0.431 ± 0.092		0.906	0.423 ± 0.129	0.470 ± 0.136		0.108	0.425 ± 0.130	0.507 ± 0.167		0.049
IgG	0.662 ± 0.377	0.477 ± 0.187		0.082	0.476 ± 0.220	0.475 ± 0.249		0.569	0.544 ± 0.224	0.568 ± 0.261		0.803
IgM	1.534 ± 0.254	1.780 ± 0.268		0.005	1.738 ± 0.214	1.650 ± 0.346		0.437	1.734 ± 0.251	1.755 ± 0.185		0.969
IgA	0.877 ± 0.318	0.874 ± 0.317		0.973	0.951 ± 0.382	0.918 ± 0.426		0.629	0.959 ± 0.471	1.082 ± 0.543		0.366
IgE	0.224 ± 0.095	0.148 ± 0.058		0.003	0.158 ± 0.067	0.167 ± 0.072		0.562	0.158 ± 0.056	0.175 ± 0.080		0.432
(**B**) *Pseudoterranova decipiens*												
	**Healthy**				**Ulcerative Colitis**				**Crohn’s Disease**			
	**Men**	**Women**			**Men**	**Women**			**Men**	**Women**		
	**16**	**25**		***p***	**43**	**43**		***p***	**22**	**46**		***p***
IgG	0.571 ± 0.291	0.547 ± 0.302		0.521	0.495 ± 0.340	0.483 ± 0.296		0.947	0.511 ± 0.431	0.478 ± 0.283		0.839
IgA	0.637 ± 0.648	0.382 ± 0.388		0.336	0.442 ± 0.345	0.603 ± 0.608		0.847	0.436 ± 0.459	0.370 ± 0.405		0.618
IgE	0.030 ± 0.023	0.036 ± 0.024		0.433	0.034 ± 0.018	0.034 ± 0.022		0.949	0.038 ± 0.029	0.038 ± 0.023		0.718
(**C**) Sex distribution across study cohorts												
	**Healthy**		**Ulcerative Colitis**		**Healthy**		**Crohn’s** **Disease**		**Ulcerative Colitis**		**Crohn’s** **Disease**	
**Men**	16		43		16		22		43		22	
**Women**	25		43		25		46		43		46	
**Chi^2^**	1.345				0.501				4.848			
***p***	0.246				0.479				0.028			
**OR**	0.640 (0.300. 1.364)				1.338 (0.597. 3.000)				2.091 (1.080. 4.049)			

**Table A3:** (**A**) mean optical densities of anti-*Anisakis simplex* antibodies with corresponding standard deviations, stratified by smoking status (smokers vs. non smokers) in healthy controls, ulcerative colitis (UC) patients, and Crohn’s disease (CD) patients. (**B**) mean optical densities of anti-*Pseudoterranova decipiens* antibodies with corresponding standard deviations, categorized by smoking habits (smokers vs. nonsmokers) in healthy controls, UC patients, and CD patients. (**C**) comparative analysis of the numbers of smokers across the three study populations (healthy controls, UC patients, and CD patients).

(**A**) *Anisakis simplex*												
	**Healthy**				**Ulcerative Colitis**				**Crohn’s Disease**			
	**Non Smokers**	**Smokers**			**Non Smokers**	**Smokers**			**Non Smokers**	**Smokers**		
	**28**	**13**		***p***	**28**	**13**		***p***	**28**	**13**		***p***
**Igs**	0.437 ± 0.115	0.420 ± 0.131		0.691	0.437 ± 0.115	0.420 ± 0.131		0.691	0.437 ± 0.115	0.420 ± 0.131		0.691
**IgG**	0.537 ± 0.281	0.588 ± 0.317		0.453	0.537 ± 0.281	0.588 ± 0.317		0.453	0.537 ± 0.281	0.588 ± 0.317		0.453
**IgM**	1.674 ± 0.275	1.673 ± 0.307		0.994	1.674 ± 0.275	1.673 ± 0.307		0.994	1.674 ± 0.275	1.673 ± 0.307		0.994
**IgA**	0.868 ± 0.298	0.888 ± 0.368		0.857	0.868 ± 0.298	0.888 ± 0.368		0.857	0.868 ± 0.298	0.888 ± 0.368		0.857
**IgE**	0.171 ± 0.075	0.195 ± 0.098		0.399	0.171 ± 0.075	0.195 ± 0.098		0.399	0.171 ± 0.075	0.195 ± 0.098		0.399
(**B**) *Pseudoterranova decipiens*												
	**Healthy**				**Ulcerative Colitis**				**Crohn’s Disease**			
	**Non Smokers**	**Smokers**			**Non Smokers**	**Smokers**			**Non Smokers**	**Smokers**		
	**28**	**13**		***p***	**28**	**13**		***p***	**28**	**13**		***p***
**IgG**	0.565 ± 0.287	0.535 ± 0.331		0.394	0.565 ± 0.287	0.535 ± 0.331		0.394	0.565 ± 0.287	0.535 ± 0.331		0.394
**IgA**	0.428 ± 0.461	0.623 ± 0.615		0.378	0.428 ± 0.461	0.623 ± 0.615		0.378	0.428 ± 0.461	0.623 ± 0.615		0.378
**IgE**	0.032 ± 0.021	0.036 ± 0.030		0.563	0.032 ± 0.021	0.036 ± 0.030		0.563	0.032 ± 0.021	0.036 ± 0.030		0.563
(**C**) Sex distribution across study cohorts												
	**Healthy**		**Ulcerative Colitis**		**Healthy**		**Crohn’s Disease**		**Ulcerative Colitis**		**Crohn’s Disease**	
**Non Smokers**	28		62		28		43		62		43	
**Smokers**	13		24		13		25		24		25	
**Chi^2^**	0.194				0.288				1.373			
***p***	0.659				0.591				0.241			
**OR**	0.834 (0.371. 1.873)				1.252 (0.550. 2.849)				1.502 (0.759. 2.970)			

**Table A4:** Correlation analysis of anti-*Anisakis simplex* and anti-*Pseudoterranova decipiens* antibody optical density (OD) values with age in clinical cohorts. (**A**) Pearson/Spearman correlation coefficients for anti-*A. simplex* antibody OD values in healthy controls, ulcerative colitis (UC) patients, and Crohn’s disease (CD) patients. (**B**) Pearson/Spearman correlation coefficients for anti-*P. decipiens* antibody OD values in healthy controls, UC patients, and CD patients.

(**A**) *Anisakis simplex*									
	**Healthy**			**Ulcerative Colitis**			**Crohn’s Disease**		
	**Rho**	**Pearson**	***p***	**Rho**	**Pearson**	***p***	**Rho**	**Pearson**	***p***
**Igs**		−0.060	0.712		−0.201	0.064		−0.152	0.216
**IgG**	0.171		0.286	0.075		0.491	−0.152		0.217
**IgM**		−0.496	<0.001	−0.354		<0.001	−0.210		0.086
**IgA**		0.007	0.963	0.081		0.456	0.234		0.055
**IgE**		0.073	0.652		0.130	0.233	−0.273		0.024
(**B**) *Pseudoterranova decipiens*									
	**Healthy**			**Ulcerative Colitis**			**Crohn’s Disease**		
	**Rho**	**Pearson**	***p***	**Rho**	**Pearson**	***p***	**Rho**	**Pearson**	***p***
**IgG**	0.026		0.873	0.206		0.058	0.069		0.575
**IgA**	−0.180		0.259	0.341		0.001	0.115		0.350
**IgE**		−0.009	0.954		0.118	0.283	−0.018		0.886

**Table A5:** Mean optical density (OD) values and standard deviations for anti-*Anisakis simplex* (**A**) and anti-*Pseudoterranova decipiens* (**B**) antibodies stratified by Montreal classification phenotypes in ulcerative colitis (UC) patients (**1**) and Crohn’s disease (CD) patients (**2**).

(**1**) Ulcerative colitis						
(**A1**) *Anisakis simplex*						
	**Proctitis**	**Left**	**Total**	**Proc vs. Left**	**Proc vs. Total**	**Left vs. Total**
	**23**	**41**	**19**	***p***	***p***	***p***
**Igs**	0.448 ± 0.104	0.451 ± 0.149	0.422 ± 0.141	0.925	0.495	0.474
**IgG**	0.512 ± 0.211	0.452 ± 0.271	0.481 ± 0.180	0.049	0.658	0.198
**IgM**	1.710 ± 0.290	1.673 ± 0.311	1.700 ± 0.263	0.437	0.604	0.987
**IgA**	0.966 ± 0.358	0.901 ± 0.369	0.887 ± 0.488	0.48	0.356	0.386
**IgE**	0.171 ± 0.067	0.161 ± 0.074	0.163 ± 0.067	0.569	0.689	0.909
(**B1**) *Pseudoterranova decipiens*						
	**Proctitis**	**Left**	**Total**	**Proc vs. Left**	**Proc vs. Total**	**Left vs. Total**
	**23**	**41**	**19**	***p***	***p***	***p***
**Igs**	0.533 ± 0.341	0.505 ± 0.340	0.417 ± 0.252	0.726	0.306	0.439
**IgG**	0.445 ± 0.436	0.535 ± 0.529	0.503 ± 0.482	0.706	0.854	0.915
**IgM**	0.032 ± 0.019	0.037 ± 0.021	0.033 ± 0.017	0.326	0.901	0.42
(**2**) Crohn’s disease						
(**A2**) *Anisakis simplex*						
	**Ileal**	**Colonic**	**Ileocolonic**	**Ile vs. Col**	**Ile vs. Ileocol**	**Col vs. Ileocol**
	**9**	**19**	**30**	***p***	***p***	***p***
**Igs**	0.435 ± 0.087	0.483 ± 0.144	0.422 ± 0.141	0.362	0.522	0.844
**IgG**	0.432 ± 0.177	0.546 ± 0.246	0.481 ± 0.180	0.268	0.182	0.743
**IgM**	1.822 ± 0.247	1.755 ± 0.165	1.700 ± 0.263	0.248	0.172	0.424
**IgA**	0.885 ± 0.582	0.942 ± 0.414	0.887 ± 0.488	0.507	0.257	0.226
**IgE**	0.140 ± 0.049	0.165 ± 0.071	0.163 ± 0.067	0.431	0.301	0.943
(**B2**) *Pseudoterranova decipiens*						
	**Ileal**	**Colonic**	**Ileocolonic**	**Ile vs. Col**	**Ile vs. Ileocol**	**Col vs. Ileocol**
	**9**	**19**	**30**	***p***	***p***	***p***
**Igs**	0.331 ± 0.170	0.641 ± 0.361	0.471 ± 0.368	0.019	0.217	0.049
**IgG**	0.335 ± 0.279	0.415 ± 0.390	0.406 ± 0.504	0.605	0.894	0.378
**IgM**	0.026 ± 0.020	0.041 ± 0.025	0.039 ± 0.020	0.121	0.102	0.805

**Table A6:** Correlation coefficients of anti-*Anisakis simplex* antibodies (**A**) and anti-*Pseudoterranova decipiens* antibodies (**B**) with time from diagnosis to study enrollment of ulcerative colitis (UC) and Crohn’s disease (CD) patients.

(**A**) *Anisakis simplex*				
	**UC**		**CD**	
	**Rho**	***p***	**Rho**	***p***
**Igs**	0.060	0.587	0.117	0.341
**IgG**	0.258	0.017	−0.014	0.911
**IgM**	−0.136	0.214	−0.051	0.681
**IgA**	0.153	0.162	0.403	<0.001
**IgE**	0.283	0.009	−0.226	0.064
(**B**) *Pseudoterranova decipiens*				
	**UC**		**CD**	
	**Rho**	***p***	**Rho**	***p***
**IgG**	0.141	0.201	0.061	0.622
**IgA**	0.294	0.007	−0.046	0.707
**IgE**	0.012	0.910	−0.016	0.895

**Table A7:** Mean values and standard deviations of optical densities (OD) for anti-*Anisakis simplex* (**A**) and anti-*Pseudoterranova decipiens* (**B**) antibodies in patients with Crohn’s disease (CD), stratified by surgical intervention status.

(**A**) *Anisakis simplex*			
	**Without Surgery**	**With Surgery**	
	**42**	**26**	***p***
**Igs**	0.479 ± 0.171	0.482 ± 0.143	0.942
**IgG**	0.563 ± 0.256	0.555 ± 0.241	0.990
**IgM**	1.764 ± 0.205	1.723 ± 0.211	0.405
**IgA**	0.952 ± 0.521	1.188 ± 0.495	0.037
**IgE**	0.175 ± 0.081	0.160 ± 0.060	0.434
(**B**) *Pseudoterranova decipiens*			
	**Without Surgery**	**With Surgery**	
	**42**	**26**	***p***
**IgG**	0.442 ± 0.251	0.565 ± 0.433	0.394
**IgA**	0.325 ± 0.347	0.499 ± 0.507	0.057
**IgE**	0.038 ± 0.028	0.038 ± 0.020	0.645

**Table A8:** Mean optical density values and standard deviations for anti-*Anisakis simplex* and anti-*Pseudoterranova decipiens* antibody levels stratified by clinical disease activity according to patient-specific scoring systems for ulcerative colitis (**1**) and Crohn’s disease (**2**).

(**1**) Ulcerative colitis						
(**A1**) *Anisakis simplex*						
	**Mayo**			**Mayo Endoscopic**		
	**Remission**	**Active**		**Remission**	**Active**	
	**19**	**64**	***p***	**34**	**48**	***p***
**Igs**	0.494 ± 0.164	0.434 ± 0.122	0.089	0.458 ± 0.166	0.442 ± 0.122	0.599
**IgG**	0.515 ± 0.312	0.456 ± 0.207	0.696	0.554 ± 0.319	0.412 ± 0.207	0.020
**IgM**	1.738 ± 0.256	1.687 ± 0.301	0.491	1.609 ± 0.263	1.761 ± 0.301	0.100
**IgA**	0.975 ± 0.386	0.921 ± 0.417	0.573	1.014 ± 0.395	0.880 ± 0.417	0.143
**IgE**	0.186 ± 0.074	0.155 ± 0.068	0.096	0.188 ± 0.076	0.144 ± 0.068	0.005
(**B1**) *Pseudoterranova decipiens*						
	**Mayo**			**Mayo Endoscopic**		
	**Remission**	**Active**		**Remission**	**Active**	
	**19**	**64**	***p***	**34**	**48**	***p***
**IgG**	0.505 ± 0.320	0.481 ± 0.324	0.809	0.513 ± 0.329	0.468 ± 0.324	0.619
**IgA**	0.592 ± 0.539	0.483 ± 0.477	0.246	0.542 ± 0.550	0.489 ± 0.477	0.286
**IgE**	0.035 ± 0.020	0.034 ± 0.020	0.391	0.033 ± 0.019	0.035 ± 0.020	0.625
(**2**) Crohn’s disease						
(**A2**) *Anisakis simplex*						
	**Harvey–Bradshaw**			**SES**		
	**Remission**	**Active**		**Remission**	**Active**	
	**39**	**29**	***p***	**27**	**41**	***p***
**Igs**	0.463 ± 0.142	0.503 ± 0.182	0.318	0.461 ± 0.127	0.493 ± 0.179	0.416
**IgG**	0.541 ± 0.184	0.586 ± 0.317	0.975	0.544 ± 0.201	0.571 ± 0.277	0.900
**IgM**	1.746 ± 0.223	1.752 ± 0.187	0.687	1.755 ± 0.218	1.744 ± 0.202	0.452
**IgA**	1.040 ± 0.583	1.045 ± 0.433	0.752	1.090 ± 0.662	1.011 ± 0.408	0.764
**IgE**	0.171 ± 0.064	0.167 ± 0.086	0.714	0.156 ± 0.051	0.177 ± 0.084	0.527
(**B2**) *Pseudoterranova decipiens*						
	**Harvey–Bradshaw**			**SES**		
	**Remission**	**Active**		**Remission**	**Active**	
	**39**	**29**	***p***	**27**	**41**	***p***
**IgG**	0.459 ± 0.274	0.529 ± 0.405	0.598	0.426 ± 0.172	0.530 ± 0.405	0.759
**IgA**	0.363 ± 0.387	0.430 ± 0.466	0.438	0.439 ± 0.382	0.360 ± 0.446	0.377
**IgE**	0.039 ± 0.024	0.037 ± 0.027	0.503	0.034 ± 0.029	0.041 ± 0.023	0.136

SES: Simple Encoscopic Score.

**Table A9:** Correlation coefficients between clinical scale scores and optical density values of anti-*Anisakis simplex* (**A**) and anti-*Pseudoterranova decipiens* (**B**) antibodies in ulcerative colitis and Crohn’s disease patients.

(**A**) *Anisakis simplex*								
	**Ulcerative Colitis**				**Crohn’s Disease**			
	**Mayo**		**Mayo Endosc**		**Harvey Brad**		**SES**	
	**Rho**	***p***	**Rho**	***p***	**Rho**	***p***	**Rho**	***p***
**Igs**	−0.112	0.313	−0.037	0.739	0.106	0.406	0.135	0.276
**IgG**	−0.272	0.013	−0.328	0.003	0.064	0.617	0.097	0.434
**IgM**	0.056	0.616	0.184	0.099	−0.117	0.356	−0.069	0.582
**IgA**	−0.146	0.188	−0.165	0.139	0.041	0.746	0.088	0.481
**IgE**	−0.361	<0.001	−0.397	<0.001	0.071	0.575	0.077	0.536
(**B**) *Pseudoterranova decipiens*								
	**Ulcerative Colitis**				**Crohn’s Disease**			
	**Mayo**		**Mayo Endoscopic**		**Harvey–Bradshaw**		**SES**	
	**Rho**	***p***	**Rho**	***p***	**Rho**	***p***	**Rho**	***p***
**IgG**	−0.065	0.562	−0.066	0.557	0.043	0.734	0.136	0.272
**IgA**	−0.132	0.237	−0.104	0.355	0.124	0.328	0.051	0.685
**IgE**	0.053	0.637	0.037	0.744	−0.055	0.664	0.165	0.182

SES: Simple Encoscopic Score.

**Table A10:** Mean optical density values and standard deviations for anti-*Anisakis simplex* (**A**) and anti-*Pseudoterranova decipiens* (**B**) antibodies in ulcerative colitis and Crohn’s disease patients, stratified by C-reactive protein (**1**) and fecal calprotectin (**2**) levels.

(**1**) C-reactive protein								
(**A1**) *Anisakis simplex*								
	**Ulcerative Colitis**					**Crohn’s Disease**		
	**Normal**	**High**				**Normal**	**High**	
	**44**	**18**	***p***			**35**	**25**	***p***
**Igs**	0.453 ± 0.122	0.409 ± 0.138	0.222			0.487 ± 0.162	0.471 ± 0.171	0.589
**IgG**	0.519 ± 0.259	0.389 ± 0.156	0.075			0.549 ± 0.255	0.606 ± 0.250	0.322
**IgM**	1.698 ± 0.313	1.669 ± 0.270	0.540			1.768 ± 0.182	1.719 ± 0.254	0.793
**IgA**	0.965 ± 0.403	0.719 ± 0.287	0.017			1.011 ± 0.530	0.972 ± 0.408	0.893
**IgE**	0.176 ± 0.073	0.142 ± 0.072	0.103			0.174 ± 0.086	0.165 ± 0.059	0.669
(**B1**) *Pseudoterranova decipiens*								
	**Ulcerative Colitis**					**Crohn’s Disease**		
	**Normal**	**High**				**Normal**	**High**	
	**44**	**18**	***p***			**35**	**25**	***p***
**IgG**	0.553 ± 0.352	0.356 ± 0.231	0.026			0.492 ± 0.275	0.517 ± 0.441	0.481
**IgA**	0.472 ± 0.422	0.407 ± 0.445	0.162			0.392 ± 0.376	0.426 ± 0.516	0.514
**IgE**	0.034 ± 0.024	0.031 ± 0.015	0.514			0.037 ± 0.024	0.042 ± 0.028	0.444
(**2**) Faecal calprotectin								
(**A2**) *Anisakis simplex*								
	**Ulcerative Colitis**					**Crohn’s Disease**		
	**Normal**	**High**				**Normal**	**High**	
	**1**	**16**	***p***			**5**	**39**	***p***
**Igs**	0.430	0.408 ± 0.113	0.852			0.443 ± 0.089	0.492 ± 0.177	0.542
**IgG**	0.408	0.470 ± 0.178	0.838			0.544 ± 0.207	0.610 ± 0.285	0.618
**IgM**	0.459	1.681 ± 0.310	0.414			1.822 ± 0.101	1.744 ± 0.205	0.471
**IgA**	0.470	0.869 ± 0.396	0.221			0.882 ± 0.255	1.008 ± 0.415	0.605
**IgE**	1.957	0.170 ± 0.061	0.562			0.177 ± 0.071	0.176 ± 0.083	0.711
(**B2**) *Pseudoterranova decipiens*								
	**Ulcerative Colitis**				**Crohn’s Disease**			
	**Normal**	**High**			**Normal**		**High**	
	**1**	**16**		***p***	**5**		**39**	***p***
**IgG**	1.681	0.461 ± 0.358		0.540	0.360 ± 0.216		0.558 ± 0.385	0.229
**IgA**	1.255	0.374 ± 0.378		0.683	0.180 ± 0.126		0.428 ± 0.459	0.215
**IgE**	0.869	0.038 ± 0.022		0.672	0.027 ± 0.011		0.047 ± 0.025	0.073

**Table A11:** Spearman/Pearson’s rank correlation analyses evaluating associations between optical densities of anti-*Anisakis simplex* (**a**) and anti-*Pseudoterranova decipiens* (**b**) antibodies in ulcerative colitis (UC) and Crohn’s disease (CD) patients stratified by C-reactive protein and fecal calprotectin levels.

(**a**) *Anisakis simplex*									
	**Fecal Calprotectin**					**C-Reactive Protein**			
	**Ulcerative Colitis**			**Crohn’s Disease**		**Ulcerative Colitis**		**Crohn’s Disease**	
	**Rho**	**Pearson**	***p***	**Rho**	***p***	**Rho**	***p***	**Rho**	***p***
**Igs**		−0.053	0.841	−0.073	0.638	−0.247	0.053	−0.006	0.965
**IgG**	−0.118		0.652	0.070	0.650	−0.231	0.070	0.187	0.155
**IgM**	−0.064		0.808	−0.298	0.050	−0.110	0.394	−0.022	0.871
**IgA**	−0.248		0.337	−0.104	0.504	−0.308	0.015	0.019	0.884
**IgE**		−0.082	0.754	0.042	0.785	−0.238	0.063	0.132	0.319
(**b**) *Pseudoterranova decipiens*									
	**Fecal Calprotectin**					**C-Reactive Protein**			
	**Ulcerative Colitis**			**Crohn’s Disease**		**Ulcerative Colitis**		**Crohn’s Disease**	
	**Rho**	**Pearson**	***p***	**Rho**	***p***	**Rho**	***p***	**Rho**	***p***
**IgG**	−0.059		0.822	−0.055	0.721	−0.256	0.046	−0.020	0.881
**IgA**	0.165		0.528	−0.217	0.157	−0.137	0.293	−0.040	0.761
**IgE**		0.146	0.576	0.252	0.099	−0.039	0.768	0.092	0.490

**Table A12:** Anti-*Pseudoterranova decipiens* antibody optical density (OD) values were stratified by clinical outcomes and treatment in ulcerative colitis (UC) and Crohn’s disease (CD) patients. (**a**) mean OD values (with standard deviations) of anti-*P. decipiens* antibodies were compared between UC and CD patients categorized by overall prognosis (good vs. poor). (**b**) anti-*P. decipiens* antibody OD values (mean ± SD) were analyzed in UC and CD subgroups based on treatment regimens. (**c**) anti-*Anisakis simplex* antibody OD values (mean ± SD) were evaluated in UC and CD patients stratified by surgical prognosis (good vs. poor). Anti-*P. decipiens* antibody OD values (mean ± SD) were similarly compared between these surgical prognosis groups.

(**a**) Prognosis.									
	**Ulcerative Colitis**					**Crohn’s Disease**			
	**Good**		**Poor**			**Good**		**Poor**	
	**51**		**28**		***p***	**23**		**39**	***p***
**IgG**	0.549 ± 0.324		0.383 ± 0.288		0.004	0.469 ± 0.215		0.523 ± 0.406	0.732
**IgA**	0.593 ± 0.547		0.404 ± 0.405		0.154	0.486 ± 0.489		0.350 ± 0.384	0.386
**IgE**	0.032 ± 0.020		0.035 ± 0.020		0.510	0.031 ± 0.020		0.044 ± 0.027	0.078
(**b**) Prognosis based on treatment regimens.									
	**Ulcerative Colitis**					**Crohn’s Disease**			
	**Good**		**Poor**			**Good**	**Poor**		
	**55**		**24**		***p***	**22**	**46**		***p***
**IgG**	0.551 ± 0.326		0.351 ± 0.262		0.001	0.506 ± 0.262	0.507 ± 0.408		0.392
**IgA**	0.577 ± 0.541		0.409 ± 0.404		0.258	0.478 ± 0.470	0.345 ± 0.393		0.368
**IgE**	0.033 ± 0.021		0.034 ± 0.019		0.931	0.031 ± 0.020	0.045 ± 0.029		0.052
(**c**) Surgical prognosis.									
(**c1**) *Anisakis simplex*									
	**Ulcerative Colitis**					**Crohn’s Disease**			
	**Good**		**Bad**			**Good**	**Bad**		
	**55**		**24**	***p***		**22**	**46**		***p***
**Igs**	0.437 ± 0.138		0.440 ± 0.149	0.875		0.483 ± 0.167	0.489 ± 0.128		0.902
**IgG**	0.491 ± 0.246		0.430 ± 0.184	0.746		0.559 ± 0.235	0.545 ± 0.308		0.670
**IgM**	1.674 ± 0.302		1.785 ± 0.281	0.301		1.753 ± 0.208	1.777 ± 0.165		0.856
**IgA**	0.912 ± 0.388		0.965 ± 0.473	0.890		1.006 ± 0.458	1.132 ± 0.488		0.369
**IgE**	0.170 ± 0.071		0.125 ± 0.036	0.066		0.171 ± 0.082	0.155 ± 0.037		0.814
(**c2**) *Pseudoterranova decipiens*									
		**Ulcerative Colitis**				**Crohn’s Disease**			
		**Good**	**Poor**			**Good**	**Poor**		
		**55**	**24**	***p***		**22**	**46**		***p***
**IgG**		0.503 ± 0.321	0.389 ± 0.315	0.157		0.481 ± 0.280	0.461 ± 0.315		0.611
**IgA**		0.553 ± 0.520	0.315 ± 0.335	0.189		0.388 ± 0.395	0.312 ± 0.278		0.690
**IgE**		0.033 ± 0.020	0.037 ± 0.023	0.581		0.039 ± 0.027	0.040 ± 0.021		0.792

**Table A13:** Numbers and percentages of ulcerative colitis and Crohn’s disease patients receiving each treatment.

	Ulcerative Colitis		Crohn’s Disease	
	n	%	n	%
Any Treatment	65	75.6	47	69.1
Immunosuppresor Treatment	27	31.4	41	60.3
Mesalazine	49	57.0	15	22.1
Sulfasalazine	7	8.1	3	4.4
Corticoids	19	22.1	21	30.9
Azatioprine	6	7.0	21	30.9
Anti-TNFα Treatment	6	7.0	8	11.8
